# Evaluation of a Cell-Based Potency Assay for Detection of the Potency of TrenibotulinumtoxinE^®^ (TrenibotE)

**DOI:** 10.3390/toxins18010019

**Published:** 2025-12-29

**Authors:** Yingchao Yang, Huajie Zhang, Shuo Wang, Yanhua Xue, Liyong Yuan

**Affiliations:** National Institutes for Food and Drug Control, Beijing 102629, China; yangyc@nifdc.org.cn (Y.Y.);

**Keywords:** botulinum toxin type E, CBPA, SNAP25_180_

## Abstract

(1) Background: As an innovative drug derived from botulinum neurotoxin serotype E, TrenibotulinumtoxinE^®^ demonstrates a rapid onset and shorter effect. Due to concerns regarding specificity, test throughput, and animal welfare, a new cell-based potency assay (CBPA) method was developed for BoNT/E drug substance and drug product; independent evaluation of this new CBPA was required. (2) Methods: The CBPA for BoNT/E is a quantitative assay that measures the accumulated cleaved SNAP25_180_ in human neuroblastoma cells. It involves sequential culturing, differentiation of cells, and then treatment with drug products. Data were analyzed using a quadratic parallel model via statistical software. Linearity was determined using five effective concentration levels. Key assay parameters including accuracy, linearity, repeatability, intermediate precision and range were evaluated. (3) Results: The overall assay’s accuracy was 98%, and the intermediate precision was 6.3%. The coefficient of determination (R^2^) and slope were determined as 0.963 and 0.942, respectively. The root mean squared error (RMSE) was 0.057, and the intercept was 0.032 for the combined data. The repeatability was 2.4%, which is well within the acceptance criterion of ≤8%. (4) Conclusions: The evaluation was carried out within a single laboratory under controlled conditions; the new CBPA meets all acceptance criteria and can be used for BoNT/E potency determination.

## 1. Introduction

Botulinum neurotoxins (BoNTs) are synthesized by Clostridium botulinum and are the most potent known natural toxins [1,2]. Among the seven different serotypes of the toxin (A–G), serotypes A, B, E, and F cause botulism in humans, and serotypes C and D have been attributed to veterinary botulism [3]. The outbreak of human serotype E botulism was mainly associated with consuming fish and aquatic mammals [4], but most cases were found far from lakes and oceans in China [5].

The soluble N-ethylmaleimide-sensitive factor attachment protein receptor (SNARE) complex is formed by the synaptosome-associated protein of 25 kDa (SNAP-25), syntaxin, and vesicle-associated membrane protein (VAMP)/synaptobrevin, which mediates the release of acetylcholine from synaptic vesicles [6]. Serotypes A, C, and E cleave at different sites of SNAP-25, while serotypes B, D, F, and G cleave at different sites of VAMP. After the cleavage of any SNARE protein mentioned above, the formation of the SNARE complex is inhibited, resulting in the blockage of neuro-transmitter release. This leads to the classical paralytic symptoms of botulism [7,8].

Botulinum toxin A (BoNT/A) has been used for therapeutic and esthetic indications since the 1980s [9]. No other BoNT serotype product has been approved in China except six BoNT/A products. MYOBLOC^®^, a botulinum neurotoxin serotype B (BoNT/B), was approved in the United States in December 2000 for treatment of cervical dystonia and chronic sialorrhea in adults. New BoNT products, developed from other BoNT serotypes with different pharmacological properties, are being investigated for clinical use [10,11,12,13]. Among them, TrenibotulinumtoxinE^®^ (trenibotE), a botulinum neurotoxin serotype E (BoNT/E), was developed for patients who are seeking neurotoxin treatment with a rapid onset and short effect [14].

The potency assays approved in China for neurotoxin products are the mouse LD_50_ bioassay (mLD_50_) and cell-based potency assay (CBPA). The mLD_50_ is a classic method for assaying the potency of neurotoxin products. According to previous papers [15,16,17,18], cell-based assays showed a strong potential to replace MBAs (mouse-based assays) in BoNT potency determination. In 2023, we validated the CBPA method developed by AbbVie which is equivalent to mLD_50_ and proved its compliance with the requirements in ICH Q2 and Chinese Pharmacopoeia. The replacement of mLD_50_ with CBPA is well justified in terms of ensuring safety and efficacy. The CBPA method for BOTOX^®^ then received approval from NMPA, China [19].

For multiple reasons, the CBPA method has high specificity, allowing it to be applied only to a single specific serotype toxin. Firstly, the active sites differ between serotypes. BoNT/A cuts SNAP25 between residues 197 and 198, whereas BoNT/E cuts SNAP25 at residues 180–181 [20,21]. Secondly, due to the difference in active sites, the ELISA for quantitative potency detection of toxins must employ different antibodies to specifically detect the degradations of BoNT/A to SNAP25_197_ and BoNT/E to SNAP25_180_. Finally, although the same cell line was used in CBPA for both serotypes, their sensitivity to BoNT/A and BoNT/E is different, requiring different durations for the toxin treatment and internalization process.

Therefore, due to this high serotype specificity stemming from distinct molecular mechanisms and assay conditions, it becomes necessary to develop a new CBPA specifically for AGN-151586. In an early stage of AGN-151586 development, potency was assessed using the mLD_50_ method. However, to comply with the principle of reduction, replacement, and refinement (the 3-Rs) in animal testing, the mLD_50_ method was not selected as the developed drug product potency assay by AbbVie. This decision was based on considerations related to animal welfare, assay effectiveness, and test throughput. The CBPA method was successfully developed for AGN-151586, and cross-validation between mLD_50_ and CBPA was completed and confirmed their equivalence by AbbVie (Table S5). The cross-validation also demonstrated a strong correlation between the predicted and experimentally observed mLD_50_ values for BoNT/E, confirming the model’s predictive accuracy and reliability for this serotype. Method evaluation from an independent third party is required before Biologics License Application (BLA) of CBPA for AGN-151586.

To be in accordance with General Chapter 9401 of ChP 2025, Principle Guidelines for the Validation of Biological Activity/Potency Assay for Biological Products, the validation of the CBPA method included the determination of assay parameters such as accuracy, linearity, repeatability, intermediate precision, and range. Lot 13858WP-01 of the working reference standard (WPRS) was used as both the reference standard and test sample in this study. The CBPA method was considered validated when the parameters conformed to the established criteria.

## 2. Results

### 2.1. Accuracy

A working potency reference standard, lot 13858WP-01 (nominal potency of 1400 U/vial), was used. It was manufactured and reconstituted for CBPAs in medium as commercial TrenibotulinumtoxinE^®^ lots. The experimental reference standard and test sample were WPRS lot 13858WP-01. It was performed at five target potency levels (50%, 75%, 100%, 125%, and 150%) by two analysts on different testing days, and a minimum of *n* = 3 CBPA results for each potency level were tested. The criterion was within 85% to 115% recovery of the target potency level.

Equation (1) was used to calculate accuracy, where the measured relative potency value obtained from CBPA test was divided by the nominal relative potency value multiplied by 100% to obtain the accuracy percentage.

The overall accuracy was generated by calculating the mean accuracy by adding the accuracy results at each potency level and dividing by the number of potency levels.

As shown in Table 1, below, the overall accuracy was 98%, and mean accuracy was 104% for the 50% potency level, 100% for the 75% potency level, 93% for the 100% potency level (repeatability data not included), 98% for the 125% potency level, and 98% for the 150% potency level. All accuracies fall within the acceptance criteria of 85–115%.

### 2.2. Intermediate Precision

Intermediate precision was evaluated by calculating the mean relative potency, standard deviation (SD), coefficient of variation (CV), and overall CV for each potency level (50%, 75%, 100%, 125%, and 150%) using Equation (2).

Two analysts independently tested WPRS lot 13858WP-01 at five relative potency levels, in different testing sessions. The mean relative potency values and the standard deviations were used to calculate the CV for each potency level.

As summarized in Table 2, the CV was 7.57% at 50% relative potency level, 4.20% at 75%, 6.96% at 100%, 6.99% at 125%, and 1.36% at 150%. All results met intermediate precision acceptance criteria of CV ≤ 8% at 100% relative potency level and CV ≤ 13% at all other levels. The overall CV was 6.28%.

### 2.3. Linearity

Linearity was evaluated using accuracy and intermediate precision data. The criterion for linearity is that the slope of the plot of expected vs. measured values should fall between ≥0.80 and ≤1.25, with R^2^ ≥ 0.90. The results of Y-intercept and RMSE (root mean square error) were reported.

**Figure 1:**
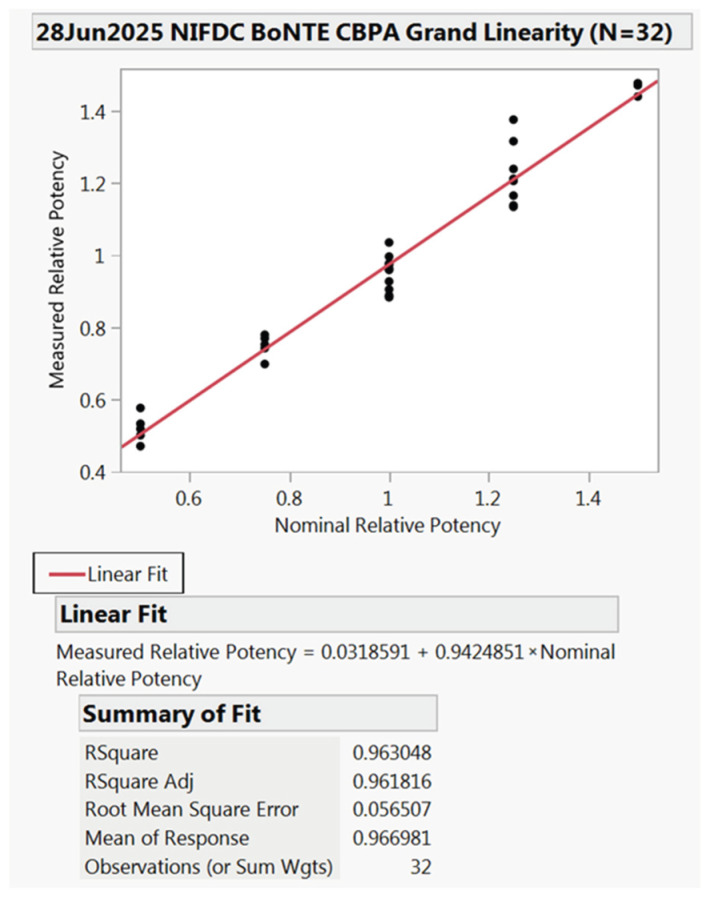
Linearity results of CBPA. (The dots were 50%, 75%, 100%, 125%, and 150% nominal relative potency, respectively).

As summarized in Figure 1, the slope of the plot of measured potency against expected potency was 0.942, and the R^2^ value was 0.963, both meeting the criteria. The Y-intercept and RMSE were 0.032 and 0.057, respectively.

### 2.4. Repeatability

Repeatability was assessed at the nominal concentration level of 100% by a single analyst using the same set of equipment, critical reagents, and materials, with a minimum of *N* = 6 replicates. Repeatability was determined by calculating the coefficient of variation (CV) of the relative potencies at the 100% level.

As shown in Table 3 below, the assay repeatability was 2.38%, which met the acceptance criterion of ≤8%.

### 2.5. Range

The range of the analytical procedure is the interval between the upper and lower concentration (or amounts) of analyte, for which it has been demonstrated that the analytical procedure exhibits suitable accuracy, precision, and linearity.

In this study, the range was evaluated at 50%, 75%, 100%, 125%, and 150% nominal relative potency levels of 135858WP-01 against 13858WP-01 (self-to-self comparison).

The results show that all potency levels meet the acceptance criteria for accuracy and linearity, confirming that the CBPA method is suitable for quantification of BoNT/E samples within the range of 50% to 150% of label claim.

## 3. Discussion

The development of a cell-based potency assay (CBPA) for BoNT/E, such as AGN-151586, addresses a distinct need within neurotoxin research. Unlike the well-characterized and widely used BoNT/A, BoNT/E exhibits a faster onset and shorter duration of action, attributed to its different molecular mechanism. The assay presented here is therefore not a mere adaptation of a BoNT/A protocol but a necessary development to support the specific quality control and potency assurance of BoNT/E.

The CBPA method for onabotulinumtoxinA obtained regulatory approval in October 2024 from NMPA, China. It was first approved by the United States Food and Drug Administration and subsequently by the European Union. Considering the limited number of sample batches that can be tested by mLD_50_ in parallel and the rapid expansion of the Chinese market for neurotoxin products, the CBPA method for onabotulinumtoxinA has a significant potential advantage in meeting the growing demand for product testing. The mLD_50_ assay, as the standard method for BoNT/A potency testing, was reserved on the Standard of Quality for onabotulinumtoxinA.

Cell-based assays (CBAs) were developed for neurotoxins, such as incobotulinumtoxinA (Xeomin^®^) [22] and abobotulinumtoxinA (Dysport^®^, Azzalure^®^; ALLUZIENCE^®^) [15]. For these CBA methods, method transfer, validation, and cross-validation with mLD_50_ in China has not yet been conducted, so the potency test for these products in China is still mLD_50_.

Due to quicker onset and shorter effect, BoNT/E was a promising drug candidate when compared to BoNT/A and BoNT/B [23,24,25]. In addition to the sensitivity of CBPA to BoNT/E, the short exposure time of cells to the toxin and excipients in the formulation of the BoNT/E drug product, such as surfactants, and protein activity protectants, can lead to cytotoxicity, thereby affecting the cellular state. Cytotoxic effects, attributed to excipient-induced osmotic pressure, were indeed observed during assay development and were mitigated by incorporating a critical dilution step into the validated protocol. This ensures the final readout is specific to SNAP-25 cleavage.

The AGN-151586 CBPA overcomes the challenges mentioned above and measures the potency by assessing the level of accumulated cleaved SNAP25_180_ in human neuro-blastoma cells (BB10). The studies presented in this method validation report detail the experiments, and the results met the pre-determined acceptance criteria outlined in the validation protocol discussed with AbbVie and determined by the National Institutes for Food and Drug Control, China.

The mLD_50_ assay was used for release and stability testing of drug substance and drug product from early development to phase 3 by AbbVie. The validation for both DP and DS is described in Tables S3 and S4. The acceptance criteria for accuracy of mLD_50_ validation for drug product failed at the 150% level during the initial validation experiments, as shown in Table S4. The assay passed all acceptance criteria outlined in the validation protocol at the 145% level and was found to be valid within the 50–145% range. The 150% claim is supported by the CBPA’s internal validation data rather than by direct comparability with the mLD_50_ at that specific level. The mLD_50_ was later replaced with a cell-based potency assay in the spirit of 3-Rs principles in animal experimentation to eliminate the need for animal testing.

The equivalence between CBPA and mLD_50_ methods was demonstrated by measuring the potency of four drug substances and four drug product batches by both assays and calculating a ratio for potency determined by CBPA relative to that determined by mLD_50_. CBPA: mLD_50_ ratio was calculated for each DS and DP batch and the results are depicted in Table S5. The overall geometric mean potency ratio for CBPA:mLD_50_ was found to be 0.98 with 90% confidence interval ranging overall from 0.96 to 1.00. The head-to-head comparison (Table S5) shows that the overall mean potency determined by CBPA is consistent with that determined by mLD_50_, and the variability of the CBPA method is consistent and shows only minor differences to that of the mLD_50_ method.

The stability-indicating capability of the CBPA and mLD_50_ methods was assessed by testing temperature-stressed DS, ultraviolet (UV) light-stressed DP and cool white light (CWL)-stressed DP, as well as unstressed control samples as comparators. The CBPA was demonstrated to be stability-indicating for all three stress conditions. The mLD_50_ was demonstrated to be stability-indicating for UV- and CWL-stressed samples but not for the temperature-stressed sample. The CBPA detected an earlier and greater loss of potency under stress conditions, which is consistent with its ability to measure specific steps in the mechanism of action that are compromised early in the product’s degradation pathway. This makes it a valuable tool for monitoring product stability (Table S5).

The CBPA method was found to be equivalent to mLD_50_ and sensitive to the potency changes of TrenibotulinumtoxinE^®^, therefore, it was deemed to be a suitable orthogonal method to mLD_50_ and was adopted to assess the potency of TrenibotulinumtoxinE^®^ which eliminates the necessity for animal testing.

The evaluation for CBPA used a single reference lot and was performed by a single laboratory. There was no inter-lot, inter-lab, or inter-operator variability testing. The circular nature of the validation design may also be a limitation. The experimental parameters, such as cell seeding density, exact incubation times, and reagent handling, are potential sources of variation in a cell-based assay. The fact that we did not intentionally vary them is a limitation of the current study.

Obtaining accurate potency results for BoNT/E using the mLD_50_ assay is challenging compared to BoNT/A, often requiring higher doses and more animals to achieve a measurable response without severe toxicity [10,26,27]. This is attributable to key pharmacological differences [28,29,30], including BoNT/E’s distinct receptor isoform specificity [11,25,31,32], its requirement for SV2 glycosylation [33], and its more transient intracellular activity. These factors collectively increase the workload and animal requirement for the mLD_50_, underscoring the specific utility of the developed CBPA. The CBPA successfully demonstrates parity with the mLD_50_ for release testing, while the cell model offers additional scientific value in studying receptor-specific interactions without the limitations of an in vivo model.

SiMa cells are sensitive to the neurotoxin of BoNT/A and BoNT/E [34]. BB10 cells, which originate from SiMa cells, are human neuroblastoma cells that show excellent performance for CBPA [19]. In a previous study, SiMa cells were incubated with BoNT/E at a series of concentrations (250–4000 mLD_50_/mL). BoNT/E test concentrations that were previously calibrated using mLD_50_ show a concentration-dependent increase in the level of cleaved SNAP25 [35]. This finding is consistent with the results obtained in the current study, which used a range of 700–2100 mLD_50_/mL.

Human-induced pluripotent stem cell (hiPSC)-derived neurons, such as GABA or motor, were seeded in coated 96-well plates and cultured for several days. The cells were then treated with BoNT/A toxin, and the level of cleaved SNAP25 was detected using Western blot or ELISA [36].

We have also applied hiPSC-derived forebrain neurons in detecting the potency of BoNT/A and BoNT/E. We have obtained some results and will perform method validation and cross-validation with mLD_50_ starting from next year.

A new clinical use for BoNT is the mixture of different serotype toxins, such as BoNT/A and BoNT/E, as a new type of drug for joint use. This will be a new domain for BoNT toxin and will also require the CBPA method to discover the optimal formula and usage plan in the near future.

## 4. Conclusions

This study successfully evaluated a cell-based potency assay for AGN-151586 Drug Product, confirming its accuracy, linearity, repeatability, intermediate precision and range under controlled conditions. The evaluated CBPA method is therefore suitable for the drug release testing of TrenibotulinumtoxinE^®^.

A primary limitation is that the validation was conducted within a single laboratory, and future work should involve a multi-center collaborative study to confirm transferability and robustness. This reliable assay establishes a critical quality control tool for the commercial lifecycle of this biologic therapeutic.

## 5. Materials and Methods

### 5.1. Sample Preparations

Five different potency level samples were reconstituted and prepared by using WPRS lot 13858WP-01. Its assigned potency is 1301 U/vial, its potency 95% Confidence Interval is 1292~1311 U/vial. The assigned protein content is 12.45 ng/vial, its assigned protein content 95% Confidence Interval is 11.8–13.1 ng/vial. It was manufactured and released by Allergan Pharmaceuticals Ireland. The assigned potency of 1301 U/vial falls well within the predefined acceptance criteria (80–120% of nominal potency) required for product quality control and batch release, confirming its consistency and suitability for use.

The production process is as follows: (1) raw material weighing for Bulk Excipient Solution (BES); (2) BES compounding and mixing; (3) bioburden reduction filtration into receiving bag; (4) transfer of BES into biotainer; (5) DS addition to BES and mixing; (6) sterile filtration into flexboy bag; (7) aseptic filling and partial stoppering; (8) lyophilization and stoppering; (9) crimping; (10) visual inspection.

The BoNT/E Reference Standard is a Drug Product lot qualified as a two-tiered reference standard. AGN-151586 Working Potency Reference Standard (13858WP-01) was the secondary standard product and qualified by the first level standard product. The first level standard product was qualified by mLD_50_.

Following reconstitution, each sample was subjected to four 1.3-fold serial dilutions (Figure S1) using an appropriate dilution medium to generate a series spanning five levels in accordance with the data in Table S2. The four 1.3-fold dilutions were chosen because the 1.3-fold (≈30%) increment is narrow enough to capture the steep slope of the cellular response to BoNT/E with high resolution, minimizing interpolation error.

A plate layout was used to determine the relative potency for accuracy assessment. A single analyst performed at the 100% nominal level within a single experiment for repeatability (Figure S2).

### 5.2. The CBPA Methodology [16]

#### 5.2.1. Cell Growth and Differentiation (Days 1–8)

After thawing and initial cell culture in tissue culture flasks, the cells were transferred and cultured in collagen I plates. Trisialoganglioside (GT1b) and neuronal supplements were added to enhance neurotoxin uptake.

#### 5.2.2. Cell Treatment and Cleavage of SNAP25_180_ (Day 9)

The RS and test articles were added to cell culture plates for 24 h. During this period, a heavy chain (HC) of neurotoxin was bound to the receptors of SV2 on the surface of neuro cells and was internalized, and the cleavage domain of the light chain (LC) was translocated into the cytosol, where it cleaved SNAP25 between amino acids 180 and 181.

#### 5.2.3. Cells Lysis and Quantification of SNAP25_180_ (Day 10)

After neurotoxin treatment, a sandwich enzyme-linked immunosorbent assay (ELISA) was used to quantify the cleaved SNAP25_180_ in the supernatant of lysed cells. This method utilizes a monoclonal antibody to capture both cleaved and un-cleaved SNAP25, along with a monoclonal antibody HRP-40E6 (conjugated with horseradish peroxidase (HRP)) with specific detection of SNAP25_180_. After stimulation with hydrogen peroxide substrate, the antibody of HRP-40E6 generated a transient luminescent signal and was measured using the Synergy Neo plate reader (Agilent, Santa Clara, CA, USA).

#### 5.2.4. Data Analysis

Data were analyzed using a quadratic parallel line model in JMP 18 software. This model was selected based on superior goodness-of-fit to the non-linear cellular response, and the parallelism assumption was statistically verified (F-test, *p* > 0.05) for accurate potency estimation. The raw data from both standard and test sample were analyzed and the potency of the test sample was calculated relative to the standard.

Passage 19 (P19) and Passage 20 (P20) from BB10 cell lines were used during the stage of method validation.

### 5.3. Method Validation

A total of five tests were designed and conducted on a weekly basis. Two analysts independently tested all potency levels of AGN-151586 once using the CBPA method. This yielded twelve measurements per potency level across the four-week period, allowing for validation of the accuracy, linearity, range, and intermediate precision. In each test, both analysts used one 96-well plate to measure responses, and a reportable potency result for each potency level was generated. Twelve potency results should be obtained by each analyst, and a minimum of three valid results for each potency level are required for analysis.

In the repeatability test, a single analyst with two plates performed the assay at the nominal concentration level of 100%, which should generate six sets of data. A minimum of six valid data points per potency level were required for data analysis.

The reference standards were reconstituted and prepared at 50%, 75%, 100%, 125%, and 150% of the labeled potency, serving as test samples. The study execution is provided in Figure S2.

(1) $$\mathrm{Accuracy}\;\left(\%\right)=\frac{\mathrm{Measured}\;\mathrm{Relative}\;\mathrm{Potency}\;\mathrm{Value}\;}{\mathrm{Nominal}\;\mathrm{Relative}\;\mathrm{Potency}\;\mathrm{Value}\;}\times 100\%$$

CV = (Standard Deviation/Mean) × 100%(2)

## Figures and Tables

**Table 1:** Accuracy results of CBPA.

Nominal Relative Potency (%)	Measured Relative Potency	Accuracy (%)	Mean Accuracy (%)	% CV (Precision per Level)	Overall Accuracy	Overall Intermediate Precision (%CV)
50 (*n* = 5) ^a^	0.500	100	104	7.5	98	5.4
	0.532	107				
	0.518	104				
	0.576	115				
	0.470	94				
75 (*n* = 5) ^a^	0.769	103	100	4.3		
	0.698	93				
	0.779	104				
	0.752	100				
	0.742	99				
100 (*n* = 5) ^a^	0.905	91	93	6.9		
	0.996	100				
	0.927	93				
	0.883	88				
	1.035	104				
125 (*n* = 8) ^a^	1.134	91	98	6.9		
	1.165	93				
	1.212	97				
	1.376	110				
	1.316	105				
	1.138	91				
	1.239	99				
	1.206	96				
150 (*n* = 3) ^a^	1.471	98	98	1.2		
	1.440	96				
	1.477	98				

^a^ represents the number of CBPA results.

**Table 2:** Intermediate precision results of CBPA.

Parameter	Relative Potency Level				
	50%	75%	100%	125%	150%
Mean Relative Potency (%)	51.9	74.8	94.8	122.3	146.3
Standard Deviation	0.039	0.031	0.047	0.086	0.020
CV (%)	7.57	4.20	6.96	6.99	1.369
Acceptance Criteria	≤13%	≤13%	≤8%	≤13%	≤13%
Overall CV (%)			6.28		

**Table 3:** Repeatability results of CBPA.

Potency Level	RS and TS	Measured Relative Potency	Recovery [%]	Mean Recovery (%)	Standard Deviation	CV%
100%	13858WP-01	0.996	100	97	0.023	2.38
		0.973	97			
		0.927	93			
		0.963	96			
		0.959	96			
		0.977	98			

## Data Availability

The original contributions presented in this study are included in the article/Supplementary Materials. Further inquiries can be directed to the corresponding author.

## Supplementary Materials

The following supporting information can be downloaded at: https://www.mdpi.com/article/10.3390/toxins18010019/s1, Figure S1: Plate layout for sample dilution; Figure S2: Plate layout for the validation of CBPA; Table S1: Reagents used in the validation study; Table S2: Preparation of samples at five nominal relative potency levels; Table S3: Summary of mLD_50_ Validation Results for DS; Table S4: Summary of mLD_50_ Validation Results for DP; Table S5: Summary of Characteristics Assessed for Cross-Validation.

## Abbreviations

The following abbreviations are used in this manuscript:

BoNT/A	Botulinum Toxin Type A
BoNT/E	Botulinum Toxin Type E
CBPA	Cell-Based Potency Assay
RS	Reference Standard
LD_50_	Median lethal dose
3-Rs	Reduction Replacement Refinement
