# Supplementary material for: Evaluation of a Cell-Based Potency Assay for Detection of the Potency of TrenibotulinumtoxinE® (TrenibotE)
Source: Toxins (Basel). 2025 Dec 29;18(1):19. doi: 10.3390/toxins18010019 (PMC12846514; doi:10.3390/toxins18010019)
Supplement: Supplementary file 1 [file toxins-18-00019-s001.zip › toxins-4064436-supplementary.pdf]

Supplementary Materials: Evaluation of a Cell-Based Potency Assay for Detection of the Potency of TrenibotulinumtoxinE® (TrenibotE)

Yingchao Yang, Huajie Zhang, Shuo Wang, Yanhua Xue and Liyong Yuan

Table S1. Reagents used in the validation study.

Reagent	Application	Lot Number
Rabbit monoclonal antibody against N-terminal of SNAP25	Coating antibody	111167-3
HRP-conjugated VHH anti-SNAP25/180 antibody	Detection Ab	AR24001
BB10 Cell bank	Cell line	min-iBB10WCB045A

Table S2. Preparation of samples at five nominal relative potency levels.

Nominal Relative Potency	U/vial	Reconstitution Medium Volume (mL/vial)	Reconstituted Potency Concentration (U/mL)	Top Potency Concentration (U/mL)	Dilution Medium Volume (mL)	Reconstituted Sample Needed (mL)
150%	1400	2	700	700	0	2.0
125%	1400	2	700	583.3	0.3	1.5
100%	1400	2	700	466.7	0.75	1.5
75%	1400	2	700	350	1.5	1.5
50%	1400	2	700	233.3	2.0	1.0

Table S3. Summary of mLD_{50} Validation Results for DS.

Parameter	Experimental Design	Acceptance Criteria	Relative Potency Level	Reportable Results / Minimum Required (*n*/*n*)	Result	Acceptance Criteria (Pass/Fail)
Accuracy	For individual recovery: Same DS target potency in independent experiments, at 100% concentration level (*n*=6)	Individual recoveries within 80 – 125%	100	7/6	102 - 119% Mean = 110%	Pass
Intermediate Precision	Six independent replicates executed not less than two analysts, at 100%	CV ≤ 20%	100	7/6	7%	Pass
Specificity	DS Placebo without toxin	100% survival	NA	8/8	100% survival	Pass

Parameter	Experimental Design	Acceptance Criteria	Relative Potency Level	Reportable Results / Minimum Required (*n*/*n*)	Result	Acceptance Criteria (Pass/Fail)
	2x Placebo with toxin	Recovery within 80 – 125% of the mean value. Dose response curve is similar with p-value of parallelism test parameter not being significant.	100	4/4	106%	Pass
Stability Indicating Property	Cool white light stressed DS sample and DS control sample, each tested to *n*=6.	Mean potency for results of the stressed sample (*n*=6) must be lower by statistically significant amount compared to results of control sample (*n*=6); $p<0.05$ by Student's t-test	100	6/6	Mean potencies: 56% for stressed sample, 97% for control sample, p-value = 7.6 E-05	Pass

Table S4. Summary of mLD_{50} Validation Results for DP.

Parameter	Experimental Design	Acceptance Criteria (corresponding to 80-125% of the respective potency level)	Relative Potency Level %	Reportable Results / Minimum Required (*n*/*n*)	Result, Range and Mean	Acceptance Criteria met
Accuracy	Percent recovery determined across six DP target concentrations<br><br>50%, 75%, 100%, 125%, 135%, and 145% from six independent determinations.	40-63%	50	6/6	50 – 58% Mean=51 %	Pass
		60-94%	75	6/6	69 - 80% Mean=74 %	Pass
		80-125%	100	8/6	88 – 113% Mean=97 %	Pass
		100-156%	125	6/6	103 - 139% Mean=122%	Pass
		108-169%	135	6/6	117 - 155% Mean=137%	Pass
		116-181%	145	6/6	130 - 149% Mean=141%	Pass

		120-188%	150	6/6	109 - 149% Mean=133%	Fail [a]
		Slope of the linear regression within 0.80 – 1.25	50 - 145	38/36	Slope=0.98	Pass
Repeatability	Six independent replicates executed at the 100% relative potency level	CV ≤ 20%	100	6/6	5%	Pass
Linearity	six DP target concentrations (at 50%, 75%, 100%, 125%, 135%, and 145% potency levels) with n=6 for each concentration level.	$R^2 \geq 0.90$	50 - 145	38/36	$R^2 = 0.94$	Pass
Range	Linearity, accuracy and intermediate precision was used to assess the range	The range in which the method satisfies the validation criteria for accuracy, intermediate precision, and linearity	50 - 145	38/36	Satisfied	Pass
Specificity	DP Placebo without toxin (n=8)	Potency recovery within 80 - 125%.	NA	8/8	100% survival	Pass
	2x Placebo with toxin (n=4)	Dose response curve similarity as defined in procedure.	100	4/4	97%	Pass

[a] Results at the initially proposed upper limit of 150% were variable and failed to meet the acceptance criteria for accuracy. Therefore, an approved plan was made to test 135% and 145%, as an alternative upper limit for range.

Table S5. Summary of Characteristics Assessed for Cross-Validation.

Characteristic	Test Article	Mean Potency Recovery		CBPA: mLD_{50} Ratio	Acceptance Criteria
		CBPA	mLD_{50}		
**Equivalence**	**DS BIU2202**	**1.06 (*n*=22)**	1.04 (*n*=27)	1.02	(1) The mean CBPA: mLD_{50} overall potency ratio falls within 0.90 to 1.11.<br><br>(2) The 90% confidence interval of the mean CBPA: mLD_{50} overall accuracy ratio falls within 0.85 to 1.18.
	DS BIU2203	0.98 (*n*=4)	0.97 (*n*=28)	1.01	
	DS BIX2204	1.03 (*n*=4)	1.03 (*n*=23)	1.00	
	DS BIX2205	1.02 (*n*=4)	0.90 (*n*=23)	1.13	
	DP RS006	1.02 (*n*=16)	1.02 (*n*=12)	1.00	
	DP 586.005	0.83 (*n*=10)	0.93 (*n*=24)	0.89	
	DP 586.006	0.86 (*n*=10)	0.91 (*n*=13)	0.95	
	DP 586.007	0.85 (*n*=10)	0.95 (*n*=14)	0.89	
	Mean Potency Ratio			0.98	Pass

	90% Confidence Interval of Mean Potency Ratio	0.96, 1.00	Pass

RS006 (lot# C7369C1) was used as reference standard for all test articles.

[a]: Relative potency of all stressed samples was calculated against the corresponding unstressed controls.

**Characteristic**	**Test Article**	**Stress Condition**	**Geo-mean Ratio[a] (Stress/Control)**		**Student's t-test p-value**		**Acceptance Criteria**
			**CBPA**	**mLD_{50}**	**CBPA**	**mLD_{50}**	
**Stability Indicating Property**	**DS BIU2202**	**Temp./Control**	0.84 (*n*=4, 9)	0.95 (*n*=7, 14)	< 0.0001 Pass	0.11 Fail	Statistically significant decrease in potency compared to unstressed control sample; $p < 0.05$ by Student's t-test
	DP 586.008	UV/Control	0.69 (*n*=4, 5)	0.73 (*n*=6, 6)	< 0.0001 Pass	< 0.0001 Pass	
	DP 586.008	CWL/Control	0.68 (*n*=5, 7)	0.67 (*n*=6, 7)	< 0.0001 Pass	< 0.0001 Pass	

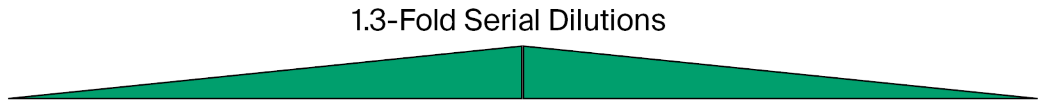

	1	2	3	4	5	6	7	8	9	10	11	12
A	NC	NC	NC	NC	NC	NC	NC	NC	NC	NC	NC	NC
B	NC	RS	RS	RS	RS	RS	S2	S2	S2	S2	S2	NC
C	NC	S1	S1	S1	S1	S1	S3	S3	S3	S3	S3	NC
D	NC	RS	RS	RS	RS	RS	S2	S2	S2	S2	S2	NC
E	NC	S1	S1	S1	S1	S1	S3	S3	S3	S3	S3	NC
F	NC	RS	RS	RS	RS	RS	S2	S2	S2	S2	S2	NC
G	NC	S1	S1	S1	S1	S1	S3	S3	S3	S3	S3	NC
H	NC	NC	NC	NC	NC	NC	NC	NC	NC	NC	NC	NC

NC: negative control; RS: reference standard; S1: sample one; S2: sample two; S3: sample three.

Figure S1. Plate layout for sample dilution.

The first week

	1	2	3	4	5	6	7	8	9	10	11	12
A	NC	NC	NC	NC	NC	NC	NC	NC	NC	NC	NC	NC
B	NC	RS	RS	RS	RS	RS	75%	75%	75%	75%	75%	NC
C	NC	50%	50%	50%	50%	50%	100%	100%	100%	100%	100%	NC
D	NC	RS	RS	RS	RS	RS	75%	75%	75%	75%	75%	NC
E	NC	50%	50%	50%	50%	50%	100%	100%	100%	100%	100%	NC
F	NC	RS	RS	RS	RS	RS	75%	75%	75%	75%	75%	NC
G	NC	50%	50%	50%	50%	50%	100%	100%	100%	100%	100%	NC
H	NC	NC	NC	NC	NC	NC	NC	NC	NC	NC	NC	NC

NC: negative control.

The second week

	1	2	3	4	5	6	7	8	9	10	11	12
A	NC	NC	NC	NC	NC	NC	NC	NC	NC	NC	NC	NC
B	NC	RS	RS	RS	RS	RS	150%	150%	150%	150%	150%	NC
C	NC	125%	125%	125%	125%	125%	50%	50%	50%	50%	50%	NC
D	NC	RS	RS	RS	RS	RS	150%	150%	150%	150%	150%	NC
E	NC	125%	125%	125%	125%	125%	50%	50%	50%	50%	50%	NC
F	NC	RS	RS	RS	RS	RS	150%	150%	150%	150%	150%	NC
G	NC	125%	125%	125%	125%	125%	50%	50%	50%	50%	50%	NC
H	NC	NC	NC	NC	NC	NC	NC	NC	NC	NC	NC	NC

NC: negative control.

The third week

	1	2	3	4	5	6	7	8	9	10	11	12
A	NC	NC	NC	NC	NC	NC	NC	NC	NC	NC	NC	NC
B	NC	RS	RS	RS	RS	RS	75%	75%	75%	75%	75%	NC
C	NC	50%	50%	50%	50%	50%	100%	100%	100%	100%	100%	NC
D	NC	RS	RS	RS	RS	RS	75%	75%	75%	75%	75%	NC
E	NC	50%	50%	50%	50%	50%	100%	100%	100%	100%	100%	NC
F	NC	RS	RS	RS	RS	RS	75%	75%	75%	75%	75%	NC
G	NC	50%	50%	50%	50%	50%	100%	100%	100%	100%	100%	NC
H	NC	NC	NC	NC	NC	NC	NC	NC	NC	NC	NC	NC

NC: negative control.

The fourth week

	1	2	3	4	5	6	7	8	9	10	11	12
A	NC	NC	NC	NC	NC	NC	NC	NC	NC	NC	NC	NC
B	NC	RS	RS	RS	RS	RS	150%	150%	150%	150%	150%	NC
C	NC	125%	125%	125%	125%	125%	50%	50%	50%	50%	50%	NC
D	NC	RS	RS	RS	RS	RS	150%	150%	150%	150%	150%	NC
E	NC	125%	125%	125%	125%	125%	50%	50%	50%	50%	50%	NC
F	NC	RS	RS	RS	RS	RS	150%	150%	150%	150%	150%	NC
G	NC	125%	125%	125%	125%	125%	50%	50%	50%	50%	50%	NC
H	NC	NC	NC	NC	NC	NC	NC	NC	NC	NC	NC	NC

NC: negative control.

The fifth week

	1	2	3	4	5	6	7	8	9	10	11	12
A	NC	NC	NC	NC	NC	NC	NC	NC	NC	NC	NC	NC
B	NC	RS	RS	RS	RS	RS	100%	100%	100%	100%	100%	NC
C	NC	100%	100%	100%	100%	100%	100%	100%	100%	100%	100%	NC
D	NC	RS	RS	RS	RS	RS	100%	100%	100%	100%	100%	NC
E	NC	100%	100%	100%	100%	100%	100%	100%	100%	100%	100%	NC
F	NC	RS	RS	RS	RS	RS	100%	100%	100%	100%	100%	NC
G	NC	100%	100%	100%	100%	100%	100%	100%	100%	100%	100%	NC
H	NC	NC	NC	NC	NC	NC	NC	NC	NC	NC	NC	NC

NC: negative control.

The repeatability test

	1	2	3	4	5	6	7	8	9	10	11	12
A	NC	NC	NC	NC	NC	NC	NC	NC	NC	NC	NC	NC
B	NC	RS	RS	RS	RS	RS	100%	100%	100%	100%	100%	NC
C	NC	100%	100%	100%	100%	100%	100%	100%	100%	100%	100%	NC
D	NC	RS	RS	RS	RS	RS	100%	100%	100%	100%	100%	NC
E	NC	100%	100%	100%	100%	100%	100%	100%	100%	100%	100%	NC
F	NC	RS	RS	RS	RS	RS	100%	100%	100%	100%	100%	NC
G	NC	100%	100%	100%	100%	100%	100%	100%	100%	100%	100%	NC
H	NC	NC	NC	NC	NC	NC	NC	NC	NC	NC	NC	NC

NC: negative control.

Figure S2. Plate layout for the validation of CBPA.
